# Thoughts on the new Continuing Professional Development guideline of the Royal College of Psychiatrists

**DOI:** 10.1192/bjb.2026.10239

**Published:** 2026-06

**Authors:** Itoro Udo, Adetokunbo Shangobiyi, Uchenna Njoku, Tamunoemi Eleye-Datubo, Tuoyo Awani, Foluke Odeyale

**Affiliations:** Department of Psychiatry, University of Western Ontario, London, Canada; City Clinic & Wellness Center, London, Canada; ClarityPath Research Institute, London, Canada; Addictions Services, City & Hackney Recovery Service (Turning Point), London, UK; Substance Misuse, Change Grow Live, Crewe, UK; Mental Health Services for Older Persons (MHSOP), Tees, Esk and Wear Valleys NHS Foundation Trust, Hartlepool, UK; University of Western Ontario, London, Canada; London Health Sciences Centre, London, Canada; Old Age Psychiatry, Hampshire and Isle of Wight NHS Trust, Gosport, UK

Having studied and appraised the new Continuing Professional Development guideline from the Royal College of Psychiatrists,^1^ we write to discuss our thoughts. We received the new guidelines with expectations, having felt that the previous guideline was long overdue for an update.^2^ This was principally because of changes to the way doctors continue to learn, occasioned by the COVID-19 pandemic. Many educational activities and opportunities now exist online, and we felt the previous guideline was missing sufficient detailed guidance in this area. The new guideline acknowledges the various ways in which doctors learn and the various educational activities that could be considered evidence of learning. It continues to maintain, encourage and embed the use of reflective practice in continuing professional development.^1^

We gratefully acknowledge a new change, which allows the accreditation of up to 8 h a day for continuing professional development, an increase from the previous 6 h. This is welcomed by our group, as our peer group meetings typically take about 8 h. Without the ability to accredit the extra 2 h of learning, we felt the previous guideline was limiting. We also welcome the introduction of the Population Health Based Discussion tool.^1^ This aligns continuing psychiatrist education with the current Royal College of Psychiatrists training curriculum. It will be an important tool that could help us to situate our clinical work in the larger population health agenda of our communities, thereby promoting our activities as advocates for people with mental illness. It would also be useful in appraisal and revalidation discussions.

There are areas that give us concern. The disintegration of academic and professional domains into a single non-clinical domain comes with risk for psychiatrists – if not in the short term, then certainly in the medium to long term. The prior mandatory requirements for academic and professional activities were very helpful in job planning, especially in services that are hard-pressed for consultant psychiatrists. With these explicit mandatory requirements, job plans could be negotiated to protect the relevant areas of professional development. We think the continuing professional development of psychiatrists is vulnerable to being eroded by service needs over the next 5 to 10 years; this would reduce the professional and, importantly, the academic involvement of psychiatrists in continuing development.

The new guideline could have been improved by the addition of an audit tool. The layout of this new guideline makes it more challenging to audit the operations of peer groups within the Royal College of Psychiatrists. In addition, various requirements, some mandatory, lacked an evidence base. The document could thus have been further improved by an appendix of evidence supporting some of the requirements, for example, the numbers of members in a peer group.

However, we recognise this occasional paper as a valuable tool to facilitate the maintenance of certification among practising psychiatrists.
